# Supplementary material for: Systemic Anti-Inflammatory Effects of Intravenous Lidocaine in Surgical Patients: A Systematic Review and Meta-Analysis
Source: J Clin Med. 2023 May 31;12(11):3772. doi: 10.3390/jcm12113772 (PMC10253813; doi:10.3390/jcm12113772)

Supplemental Table S1: Search Strategy.

Database	Query
PubMed	(intravenous OR infusion) AND (lidocaine OR lignocaine) AND (inflammatory markers OR inflammatory response OR cytokine response OR cytokines)
Scopus	TITLE-ABS-KEY ((intravenous OR infusion) AND (lidocaine OR lignocaine) AND (inflammatory markers OR inflammatory response OR cytokine response OR cytokines))
Web of Science	All Fields ((intravenous OR infusion) AND (lidocaine OR lignocaine) AND (inflammatory AND markers OR inflammatory AND response OR cytokine AND response OR cytokines))
Clinicaltrials.gov	(intravenous OR infusion) AND (lidocaine OR lignocaine) AND (inflammatory markers OR inflammatory response OR cytokine response OR cytokines)

Supplemental Figure S1: Risk of bias summary: review authors' judgements about each risk of bias item for each individual study.

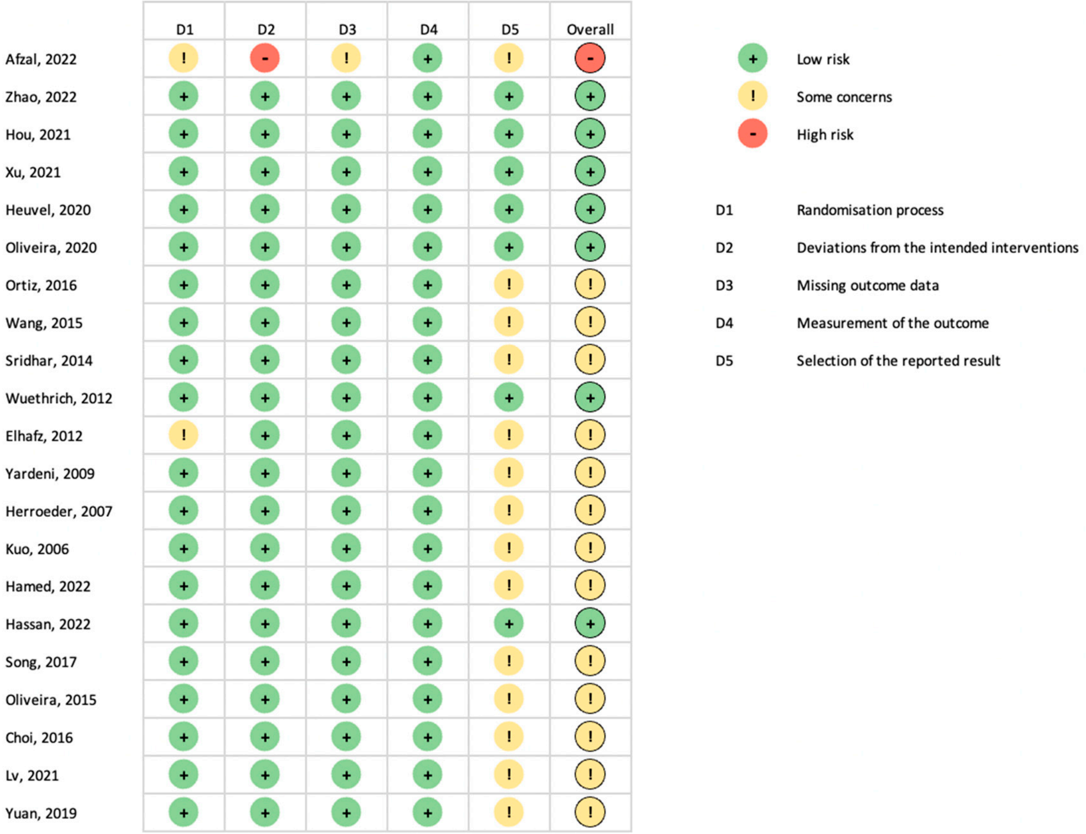

Supplement: Supplementary file 1 [file jcm-12-03772-s001.zip › jcm-2412022-supplementary.pdf]
